# Editorial

**Published:** 1844-04-06

**Authors:** 


### THE MEDICAL EXAMINER.

PHILADELPHIA, APRIL 6, 1844.

### VAGINAL HYSTEROTOMY AND CRIMINAL ABORTION.

The last number of the New York Journal of Medicine contains an account of a case in which Vaginal Hysterotomy was performed on a woman during labour, by Professor Bedford, successfully for both mother and child. The woman had borne two children previously, at full term, without difficulty, and the operation appears to have been rendered necessary in this instance in consequence of injuries done to the parts, by means employed to effect abortion, at an early stage of her pregnancy. From the account given to her medical attendants by the woman, she had *miscarried five times* previously, in consequence of drugs administered to her for the purpose, by a notorious woman calling herself "Madame Restell." About six weeks after becoming pregnant again, she called on the same "Madame," who on learning her situation, gave her some powders with directions for use; these powders did not appear to produce the desired effect. She returned again to this woman, and asked her if there was no other way to make her miscarry. "Yes," says Madame Restell, "I can probe you; but I must have my price for this operation." "What do you probe with ?" "A piece of whale-bone." "Well," thought the patient, but without expressing it, "I cannot afford to pay your price, and I will probe myself." She returned home and used the whale-bone several times; it produced considerable pain, followed by a discharge of blood.—Hence the injuries, and the operation referred to. From common report, this "Madame Restell" has been driving a brisk business in her line in New York, and from her advertisements it would seem with the greatest audacity. For a long time she contrived to escape the hands of justice; but from the exposition of her conduct by Professor Bedford in the present case, there is reason to hope she will yet reap her reward: indeed, we learn from the New York papers that the Grand Jury has already presented her to the Court, so that she will very shortly have an opportunity of proving her innocence—*if she can.* From the newspapers it also appears that several other persons have recently been convicted in that city, for similar offences. In Philadelphia, no one has been convicted of that species of murder since Chauncey: one, however, against whom a bill was found by the Grand Jury, some months ago, escaped without trial,—nobody knows how!

---

We have been favoured by Dr. Edward Hartshorne, Resident Physician of the Eastern Penitentiary of Pennsylvania, with the 15th Annual Report of that Institution. It is a highly interesting document, abounding in details concerning the health of the convicts, and the influence of the system of solitary confinement on their moral and physical condition. We regret that it came too late for the present number—it shall be particularly noticed in our next.

---

### OVARIOTOMY.

The number of cases recently published of successful extirpation of the ovaries in the human female, is calculated to induce the belief that the operation may be performed with impunity. As a proper admonition under these circumstances, we extract for our *Record* of this number, from the London Medical Gazette of January 19th, two unsuccessful cases which have recently occurred. They come from sources which must command respect, and should inspire caution. One of the greatest objections to this fearful operation is the difficulty of diagnosis—of determining the character of the tumours discovered in the abdomen, the extent of their adhesions, and various other complications, often of a serious nature, which frequently attend their development.

In the same Journal, of December 8, 1843, Mr. Meath, a highly respectable "Lecturer on Midwifery in the Manchester School of Medicine and Surgery, and one of the Honorary Surgeons to the Union Hospital," has given the particulars of a case operated on by himself, in the presence of a number of Physicians and Hospital Surgeons, which, instead of being a case of ovarian disease, turned out to be an enlarged uterus "distended with solid matter." The abdomen being opened and the tumour brought into view, the true character of the case was revealed. In that condition of things, it was determined to extirpate the organ. "The excised mass was found to consist of the whole body of the uterus, enveloping a dense adventitious structure. It was of a perfectly smooth, uniform, globular shape, presenting no trace of salient points; weighed six pounds; had a diameter from above to below of seven inches, and a circumference in the transverse direction of tweny inches." The woman died seventeen hours after the operation.

The responsibility of the mistake in this case does not rest upon Mr. Meath alone, or those connected with him in the operation, but must be ascribed to the obscurity which attends the diagnosis of such affections. The narrator tells us expressly that "after repeated examination, and most careful manipulations, by myself and colleagues (of the Hospital,) made at different times and in every variety of manner, the conclusion arrived at was the presence of an *ovarian tumor;* and it was our *unanimous* opinion that the condition of the patient, and the mobility of the tumour, made it a fair case for extirpation by the abdominal section."

Great attention has lately been given to this subject in Europe, more especially in London, and the propriety of extirpation and the best manner of accomplishing it, are points warmly discussed in the Journals. Mr. Walne has performed the operation three times successfully, and has lately "met with some unsuccessful cases." The particulars of the former have been given to the public; those of the latter have *not yet* been published. Mr. Southam, Dr. Clay, Dr. F. Bird, and Mr. Lane, have all operated successfully. Mr. C. Aston Key, "although it was long before he could bring himself to regard the extirpation of an ovarian cyst, as anything more than a successful accident, now believes that it is an operation simple in its performance, unattended with more than the usual risk of surgical operations, and called for by the hitherto intractable nature of ovarian disease." (Med. Chirurg. Rev., Jan. 1844.) Such is Mr. Key's *reasoning* on the subject—now for his *practice*. On the first of August last, he operated on "a fine-looking healthy girl," nineteen years of age, in whom the disease had commenced fifteen months previously. The operation was accompanied with no difficulties—there were no adhesions, very slight hemorrhage, and she bore the operation well,—and yet she died of peritonitis on the 5th of August, despite her "fine-looking, healthy" appearance, and all the skill and attention of Mr. Key and Dr. Lever.

The following remarks occur in an Editorial Notice of Mr. Walne's cases contained in the "British and Foreign Medical Review" of January last. Speaking of the operation: "It has been attempted in 37 cases. It has been actually performed 28 times: in 18 of these cases the patients recovered from the operation, in 10 they died: or deaths were to recoveries in a rather higher proportion than 1 to 2. Of the remaining 9 cases, in which the operation was left incomplete, either because the tumours could not be removed, or because no tumour existed, 3 terminated fatally; in the other 6 the patients recovered." The reviewer doubts whether the real results of the operation would present data so favourable, if we were in possession of all the facts on the subject. "But," he remarks, "even if we assume our present data to be correct, and that they do not at all overrate the success of ovariotomy, it may be fairly asked,—Is an operation which is followed by the death of more than 1 in 3 of those in whom it is completed, a fit subject for boasting of as a triumph of art, as constituting an era in the annals of surgery? Is human life so worthless, as to warrant the repetition of the operation, again and again, till, forsooth, we thus obtain more extended statistics?"

From the following expressions of the same writer, it is to be inferred that more *unsuccessful* cases have occurred in "the Metropolis," or some where else, than have been "permitted to see the light."

"And here we feel it our duty to animadvert, in the strongest terms, on the backwardness of those who, having so speedily published their successful cases, delay to publish their unsuccessful ones. We are unwilling to impute improper motives to these gentlemen; they may have good reasons for their silence of which we are ignorant; but the reasons must be strong indeed, to justify a proceeding fraught with such momentuous consequences to the profession, and to society at large."

